# Knowledge of human papillomavirus infection and its prevention among adolescents and parents in the greater Milan area, Northern Italy

**DOI:** 10.1186/1471-2458-10-378

**Published:** 2010-06-28

**Authors:** Claudio Pelucchi, Susanna Esposito, Carlotta Galeone, Margherita Semino, Caterina Sabatini, Irene Picciolli, Silvia Consolo, Gregorio Milani, Nicola Principi

**Affiliations:** 1 Department of Epidemiology, Istituto di Ricerche Farmacologiche Mario Negri, Milan, Italy; 2 Department of Maternal and Pediatric Sciences, Università degli Studi di Milano, Fondazione IRCCS Ca' Granda Policlinico, Milan, Italy

## Abstract

**Background:**

In order to be widely accepted by users, the implementation of a new health intervention requires them to be adequately informed about its clinical importance, benefits and risks. The aim of this study was to provide data on the knowledge of Italian adolescents and parents concerning human papillomavirus (HPV) infection and its prevention in order to allow the development of adequate training programmes.

**Methods:**

Between 2 May and 15 June 2008, we made a cross-sectional survey of 863 high school students and 2,331 parents of middle and high school students using two anonymously completed questionnaires covering the knowledge of HPV infection and related diseases, and attitudes to vaccinations. The approached schools were a convenience sample of the schools of the greater Milan area, Northern Italy.

**Results:**

More mothers than fathers were aware that HPV infection could concern their children (58% *vs *53%; p = 0.004) and were favourable towards vaccinating their children against HPV (68% *vs *65%; p = 0.03); among the students, more females than males were aware that HPV infection could concern themselves (45% *vs *26%; p < 0.001) and would undergo vaccination against HPV (68% *vs *40%; p < 0.001). The parents' propensity to vaccinate their children against HPV was significantly associated with professing the Catholic religion (odds ratio - OR = 0.61, 95% confidence interval - CI 0.46-0.82, being atheist), the gender of the offspring (OR = 1.88, 95% CI 1.53-2.30, having at least one daughter), a propensity to vaccinations in general (OR = 23.1, 95% CI 13.7-38.8), a knowledge that HPV vaccine is aimed at preventing cervical cancer (OR = 2.31, 95% CI 1.69-3.16), and an awareness that HPV could affect their own children (OR = 3.52, 95% CI 2.89-4.29). The students who were aware that HPV infection could affect themselves were more in favour of to HPV vaccination, regardless of whether they were male (OR = 5.73, 95% CI 2.85-11.5) or female (OR = 2.39, 95% CI 1.66-3.46).

**Conclusions:**

Both students and parents seem to underestimate the likelihood of HPV infection, and this is associated with a lower propensity for vaccination. This is an important indication for future training programmes concerning HPV prevention designed to increase the acceptance of HPV vaccine in families.

## Background

Persistent infection with high-risk human papillomavirus (HPV) can cause cancers of the cervix and other genital sites, anus, oral cavity and oropharynx, and possibly the larynx and skin [1-6].

In March 2008, the Italian Ministry of Health suggested to the Vaccine Committee of the Regions to start an active, free vaccination campaign against HPV as cause of cervical cancer aimed at 12-year old girls in line with the guidelines of a number of other industrialised countries [7,8]. The choice of this age was based on findings that HPV infection is almost exclusively due to sexual contact, that a large proportion of subjects are first infected during adolescence or early adulthood [9,10], and that the immune response to HPV vaccination is higher in younger subjects [11]. Moreover, the available vaccines against HPV types 16 and 18 (i.e. those associated with the highest risk of cancer) have no therapeutic effect against already established infection and HPV-associated diseases [12]. However, HPV vaccination is also indicated in older teenagers who have not yet (or have only just) started sexual activity, and might even be considered in women aged more than 25 years [11,13,14]. This explains why some Italian Regions, although maintained cervical cancer prevention as the primary purpose of prevention programme, extended the free vaccination campaign to young people aged 15 or 16 years, and others are offering older teenagers a discount on the price of HPV vaccination.

In order to be widely accepted by users, the implementation of a new health intervention requires them to be adequately informed about its clinical importance, benefits and risks. HPV vaccination is more complex than other health initiatives because the targets are young, decisions are generally made by their parents, and may not be based on reliable information. Moreover, even when younger subjects are still being followed by pediatricians, the physicians may be poorly informed about HPV-related diseases because most of these occur in adulthood [15]. In order to be successful, HPV vaccination programmes therefore need to educate not only the targets, but also their parents and healthcare providers.

This means previously acquiring adequate information concerning the knowledge and attitudes of the different subjects involved. A number of investigations carried out in different parts of the world have shown that such subjects are inadequately aware of the problem [15-22], although most of the surveys of adults' knowledge of HPV did not focus on their parental role. Furthermore, little information is available concerning Italian teenagers' and parents' knowledge of, and attitude towards HPV infection and its prevention [18,19]. Considering the importance of communication and educational material in improving vaccination coverage [23-26], the aim of this study of a large number of high school students and parents of middle and high school students was to provide some of the basic data necessary for the development of adequate training programs.

## Methods

Between 2 May and 15 June 2008, we carried out a cross-sectional survey of high school students and parents of middle and high school students in order to verify their knowledge of HPV infection and HPV-related diseases, and their attitudes to vaccinations (particularly HPV vaccine).

The survey involved two middle schools in Milan (one private and one state run), five high schools specialising in classical, linguistic or scientific studies (two private and three state run) in the greater Milan area and Varese, and one state-run technical school in Milan. The schools were a convenience sample chosen on the basis of contacts of the authors of this paper with school boards. We varied the type of schools included in the investigation in order to obtain a composite distribution of subjects reflecting the characteristics of the baseline population, with specific reference to age of the students and educational level of the parents. S.E. and N.P. had repeated contacts with boards of each school before the distribution of the questionnaires in order to assure the best compliance of teachers to the survey. After the study had been approved by the Ethics Committee of Fondazione IRCCS Ca' Granda Ospedale Maggiore Policlinico, Milan, Italy, and the boards of each school, all of the students attending the high schools were given three questionnaires: one for the student and one for each parent. Because of the age of the students attending the middle schools (10-13 years), school boards decided that only their parents had to complete a questionnaire.

The students' and parents' questionnaires were designed to collect information concerning the compilers' knowledge of HPV infection and their attitudes to vaccination. Both were to be anonymously self-completed (at home), and were accompanied by a formal letter describing the aims of the study and explaining how to complete the questionnaires. The same information was also given orally when the questionnaires were given to the students.

The questionnaires, that were prepared by a multidisciplinary group including pediatricians (SE, NP) and epidemiologists (CP, CG), were pilot-tested on a convenience sample of adolescents and parents attending the outpatient clinic of the Fondazione IRCCS Ca' Granda Ospedale Maggiore Policlinico in Milan in order to ensure clarity and ease of administration. The student questionnaire [Additional file 1] had four sections covering: 1) demographic and socio-economic characteristics such as age, race, religion, number of siblings, and the education and occupation of the parents; 2) HPV infection (its perceived dangerousness and modes of transmission) and vaccination (including preventable diseases, age at vaccination, and favourite source of vaccine information); 3) their willingness to be vaccinated against HPV; and 4) their sexual activity and attitudes to discussing sexual health issues. The parent questionnaire [Additional file 2] had five sections covering: 1) demographic and socio-economic characteristics such as age, race, religion, number and age of children, education and occupation, and family history of cancer; 2) attitudes towards vaccinations in general; 3) HPV infection and HPV vaccination; 4) their willingness to have their children vaccinated against HPV; and 5) their attitudes to discussing sexual health issues with their children and informing them about HPV infection and vaccination. The items for students were presented in an easier way, but covered aspects similar to that asked to the parents.

The students had to return the questionnaires to their teachers within a week of their distribution, and the completed questionnaires were collected from the schools by members of the research team.

The responses were analysed by means of descriptive statistics. In the case of categorical data, the gender groups (mothers vs fathers among parents and females vs males among students) were compared using the *χ*^2 ^or Fisher's exact test as appropriate. All of the analyses were two tailed, and *p *values of 0.05 or less were considered significant. Odds ratios (OR) and 95% confidence intervals (CI) were calculated in order to measure the associations between selected factors chosen on the basis of literature review and the parents' propensity to have their children vaccinated against HPV, or the students' propensity to undergo HPV vaccination themselves. The ORs were obtained by means of unconditional multiple logistic regression adjusted for: 1) age, education, gender and religion of the parents, and age and gender of their children in the case of the parents' data; or 2) age and religion of the student and education of the parents in the case of the students' data. All of the analyses were made using SAS version 9.1 (Cary, NC, USA).

## Results and discussion

A total of 3,026 questionnaires were distributed to parents (1,520 to mothers and 1,506 to fathers), 2,331 (77.0%) of which were returned with more than 70% of the questions answered. The students received a total of 1,092 questionnaires (691 given to females and 401 to males), 863 (79.0%) of which were returned with more than 70% of the questions answered. There was no difference in the response rates of the mothers and fathers, whereas female students answered significantly more frequently than male students (93.9% *vs *52.1%; p < 0.0001). There was also no significant difference in response rates and data obtained in the different schools.

Table 1 shows the distribution of the 2,331 parents who participated in the survey on the basis of selected socio-demographic characteristics. Fathers were older than mothers: respectively 44% and 23% were aged 50 years. Forty-one percent of the parents had a university degree. Twenty-three percent had only one child, 56% two children, and 21% three or more; 11% had at least one child aged <12 years, while the modal age of their children was 16-17 years (32%). Most of the parents had at least one daughter involved in the survey (72%).

**Table 1:** Distribution of parents by gender, age and selected socio-demographic characteristics

	Mothers (n = 1225)		Fathers (n = 1093)		**All (n = 2331)**^**a**^	
	**No.**	**%**	**No**.	**%**	**No.**	**%**

**Age (years)**						
<40	93	7.6	37	3.4	130	5.6
40-44	348	28.4	173	15.8	521	22.4
45-49	488	39.8	390	35.7	885	38.0
≥50	282	23.0	476	43.5	759	32.5
Missing values	14	1.2	17	1.6	36	1.5
**Mean age ± SD (years)**	46.1 ± 5.6		49.1 ± 5.6		47.5 ± 5.4	
**Education**						
Middle school diploma	176	14.4	222	20.3	398	17.1
High school diploma	564	46.0	406	37.0	973	41.7
Degree	475	38.8	462	42.4	944	40.5
Missing values	10	0.8	3	0.3	16	0.7
**No. of children**						
1	292	23.8	249	22.8	544	23.3
2	681	55.6	610	55.8	1296	55.6
3	198	16.2	180	16.5	380	16.4
≥4	50	4.1	52	4.8	103	4.4
Missing values	4	0.3	2	0.1	8	0.3
**Age of child(ren) involved in the study **^**b **^**(years)**						
<12	143	11.7	118	10.8	261	11.2
12-13	262	21.4	236	21.6	501	21.5
14-15	303	24.7	278	25.4	582	25.0
16-17	384	31.3	337	30.8	725	31.1
≥18	112	9.2	105	9.7	220	9.4
Missing values	21	1.7	19	1.7	42	1.8
**Gender of child(ren) involved in the study**						
Male	341	27.8	298	27.3	641	27.5
Female	821	67.0	719	65.8	1549	66.5
Both	57	4.7	65	5.9	122	5.2
Missing values	6	0.5	11	1.0	19	0.8

SD, standard deviation. ^a ^Thirteen parents did not answer the question concerning gender. ^b ^When more than one offspring was involved, the age of the youngest is given.

Table 2 shows the distribution of the 863 students participating in the survey on the basis of selected socio-demographic characteristics. Age distribution was similar among the males and females: the age range was 14-20 years, and the mean age 16.1 years. Most had only one sibling (55%), or were the only child in the family (22%).

**Table 2:** Distribution of students by gender, age and selected socio-demographic characteristics

	Females (n = 649)		Males (n = 209)		All **(n = 863)**^**a**^	
	**No**.	**%**	**No.**	**%**	**No.**	**%**

**Age (years)**						
14-15	231	35.6	85	40.7	317	36.7
16-17	309	47.6	92	44.0	402	46.6
≥18	107	16.5	30	14.3	137	15.9
Missing values	2	0.3	2	1.0	7	0.8
**Mean age ± SD (years)**	16.2 ± 1.44		16.0 ± 1.59		16.1 ± 1.48	
**Education (mother)**						
Middle school diploma	133	20.5	28	13.4	161	18.7
High school diploma	312	48.1	93	44.5	408	47.3
Degree	191	29.4	85	40.7	278	32.1
Missing values	13	2.0	3	1.4	16	1.9
**Education (father)**						
Middle school diploma	148	22.8	32	15.3	180	20.9
High school diploma	242	37.3	62	29.7	308	35.7
Degree	239	36.8	108	51.7	348	40.3
Missing values	20	3.1	7	3.3	27	3.1
**No. of siblings**						
0	150	23.1	41	19.6	194	22.5
1	359	55.3	115	55.0	476	55.2
2	104	16.1	38	18.2	142	16.4
≥3	36	5.5	15	7.2	51	5.9

SD, standard deviation. ^a ^Five students did not answer the question concerning gender.

Table 3 shows the distribution of the parents' and students' answers concerning their personal knowledge of HPV infection and vaccination, by gender. Mothers had ever heard of HPV more frequently than fathers (91% *vs *77%; p < 0.001) and, among the parents who were aware of HPV, the mothers were also more often aware of the dangerousness of the virus (92% *vs *88%; p = 0.004) and more informed as to how it is transmitted (94% *vs *91%; p = 0.02). Similarly, a higher proportion of female (72%) than male students (51%; p < 0.001) had ever heard of HPV but, among those who were aware of HPV, there was no significant gender-related difference in its perceived dangerousness (about 90% in both sexes) or how it is transmitted (90% *vs *92%). With reference to their knowledge of HPV vaccination, 90% of the mothers and 87% of the fathers knew that HPV vaccine is aimed at preventing cervical cancer (p = 0.02); the corresponding figures for the female and male students were 75% and 63% (p = 0.008). About 75% of the mothers, fathers and female students knew that the vaccination should be given before sexual activity begins; this decreased to 67% of the male students, although the between-gender difference among the students was not significant.

**Table 3:** Parents' and children's knowledge of HPV infection and vaccination, by gender.

	Parents					Students				
	**Mothers**		**Fathers**		**P value**^**1**^	**Females**		**Males**		**P value**^**2**^

	**No.**	**%**	**No.**	**%**		**No.**	**%**	**No.**	**%**	

**Have you ever heard of HPV?**										
No/Do not know	110	9.0	259	23.7		184	28.4	102	48.8	
Yes	1115	91.0	834	76.9	**<0.001**	465	71.6	107	51.2	**<0.001**
**Do you think HPV infection can be dangerous?**										
Yes	1026	92.0	735	88.1		421	90.5	96	89.7	
No/Only when other health conditions are present/Do not know	89	8.0	99	11.9	**0.004**	44	9.5	11	10.3	0.80
**Do you know how HPV infection is passed on?**										
Sexual intercourse	1049	94.1	762	91.4		421	90.5	99	92.5	
Wrong answers/Do not know	66	5.9	72	8.6	**0.02**	44	9.5	8	7.5	0.52
**What is the aim of HPV vaccination?**										
To prevent cervical cancer										
Yes	1005	90.1	723	86.7		350	75.3	67	62.6	
No/Do not know	110	9.9	111	13.3	**0.02**	115	24.7	40	37.4	**0.008**
To prevent a STD										
Yes	104	9.3	100	12.0		56	12.0	15	14.0	
No/Do not know	1011	90.7	734	88.0	0.06	409	88.0	92	86.0	0.58
**When should HPV vaccination be given?**^**3**^										
During the first year of life	36	3.2	25	3.0	0.77	14	3.0	3	2.8	1.00
Before sexual activity begins	852	76.4	621	74.5	0.32	351	75.5	72	67.3	0.08
After sexual activity has begun	57	5.1	48	5.8	0.53	37	8.0	8	7.5	0.87

STD, sexually transmitted disease. ^1 ^Mothers *vs *fathers. ^2 ^Females *vs *males. ^3 ^As multiple answers were allowed, each was considered a dichotomous variable (yes/no); the numbers do not add up to the total because infrequent answers are not included.

Table 4 shows the attitudes of the parents and students to vaccination (only the parents were asked questions about vaccinations in general: i.e. not specific to HPV). Both mothers (92%) and fathers (93%) were generally in favour of vaccinations. When asked which recommendations were more important when deciding whether to have their children vaccinate, they more frequently indicated the Ministry of Health (about 48% of both mother and fathers), pediatricians (50% of mothers, 43% of fathers, p = 0.001) and other physicians (28% of mothers, 24% of fathers). The mothers were more aware than the fathers that HPV infection could affect their children (58% *vs *53%, p = 0.004) and were more in favour of them being vaccinated (68% *vs *65%; p = 0.03). When these last two questions were answered by the students, 45% of females and 26% of males were aware that HPV infection could concern themselves (p < 0.001), and 68% of females and 40% of males said they would undergo HPV vaccination (p < 0.001).

**Table 4:** Parents' and students' attitude to vaccination, by gender.

	Parents					Students				
	**Mothers**		**Fathers**		**P value**^**1**^	**Females**		**Males**		**P value**^**2**^

	**No.**	**%**	**No.**	**%**		**No.**	**%**	**No.**	**%**	

**Are you in favour of vaccinations in general? **^**3,4**^										
No	97	8.0	72	6.7						
Yes	1117	92.0	1009	93.3	0.22					
**Whose recommendations are significant to you when deciding on a vaccination for your children? **^**3,5**^										
Ministry of Health	594	48.5	518	47.4	0.60					
Regional body	41	3.3	43	3.9	0.45					
Pediatrician	614	50.1	475	43.5	**0.001**					
Other physician	343	28.0	268	24.5	0.06					
Relative or friend	20	1.6	16	1.5	0.74					
Teacher	5	0.4	7	0.6	0.44					
Religious authority	6	0.5	4	0.4	0.65					
Other	78	6.4	77	7.0	0.51					
**Do you think HPV infection might concern your children (for parents)/you (for students)? **^**4**^										
Do not know	251	20.7	286	26.6		174	27.1	65	31.1	
No	255	21.0	219	20.3		179	27.9	89	42.6	
Yes	707	58.3	572	53.1	**0.004**	289	45.0	55	26.3	**<0.001**
**Would you give your children (for parents)/undergo (for students) HPV vaccination HPV? **^**4**^										
No	92	7.6	66	6.1		36	5.6	40	19.1	
Perhaps	293	24.1	308	28.7		169	26.1	86	41.1	
Yes	830	68.3	699	65.1	**0.03**	442	68.3	83	39.7	**<0.001**

^1 ^Mothers *vs *fathers. ^2 ^Female students *vs *male students. ^3 ^Question for parents only. ^4 ^The numbers do not add up to the total because of a few missing answers. ^5 ^As multiple answers were allowed, each was considered a dichotomous variable (yes/no).

Table 5 shows the associations among the parents between selected factors and their attitude to HPV vaccination for their children. There was no significant association between the propensity to have their children vaccinated and the age, gender or education of the parent, or the age of their children. The parents professing a religion other than Catholicism or who were atheists were less in favour of HPV vaccination for their children (OR = 0.61, 95% CI 0.46-0.82, compared with Catholics). Significant associations with a propensity to have their children vaccinated also included the gender of the offspring (OR = 1.88, 95% CI 1.53-2.30, having at least one daughter), a propensity to vaccinations in general (OR = 23.1, 95% CI 13.7-38.8), a knowledge that HPV vaccine is aimed at preventing cervical cancer (OR = 2.31, 95% CI 1.69-3.16), and an awareness that HPV could affect their own children (OR = 3.52, 95% CI 2.89-4.29).

**Table 5:** Associations between selected socio-demographic factors, personal beliefs and attitudes of parents to vaccinating their children against HPV.

	In favour of HPV vaccination (n = 1438)		Against or doubtful about HPV vaccination (n = 706)		**OR (95% CI) **^**2**^
	**No.**	**%**	**No.**	**%**	

**Age of parent (years)**					
<40	80	5.6	37	5.2	1 (reference)
40-44	319	22.2	172	24.4	0.85 (0.54-1.31)
45-49	565	39.3	265	37.5	1.04 (0.67-1.60)
≥50	474	33.0	232	32.9	1.05 (0.68-1.64)
**Education**					
Middle school diploma	254	17.7	122	17.3	1 (reference)
High school diploma	616	42.8	280	39.7	1.04 (0.80-1.35)
Degree	568	39.5	304	43.1	0.91 (0.69-1.19)
**Parent**					
Father	658	45.8	347	49.1	1 (reference)
Mother	780	54.2	359	50.8	1.17 (0.97-1.42)
**Religion**					
Catholic	1314	91.4	608	86.1	1 (reference)
Other/no religion	124	8.6	98	13.9	0.61 (0.46-0.82)
**Age of (youngest) child involved in the study (years)**					
<12	164	11.4	82	11.6	1 (reference)
12-14	434	30.2	218	30.9	1.04 (0.76-1.43)
≥15	840	58.4	406	57.5	0.93 (0.69-1.26)
**Gender of child(ren) involved in the study**					
Male(s) only	336	23.4	255	36.1	1 (reference)
At least one daughter	1102	76.6	451	63.9	1.88 (1.53-2.30)
**Generally in favour of vaccinations **^**3**^					
No	17	1.2	139	20.0	1 (reference)
Yes	1416	98.8	555	80.0	23.1 (13.7-38.8)
**Knows that HPV vaccine is aimed at preventing cervical cancer **^**3**^					
No/Does not know	107	8.3	91	17.0	1 (reference)
Yes	1179	91.7	444	83.0	2.31 (1.69-3.16)
**Thinks that HPV might concern own child(ren) **^**3**^					
No/Does not know	487	34.0	449	64.0	1 (reference)
Yes	946	66.0	253	36.0	3.52 (2.89-4.29)

CI, confidence interval; OR = odds ratio. ^1 ^The numbers of subjects are not the same as in Table 4 because those with missing information relating to one or more of the socio-demographic covariates in the models (n = 187; 8% of the total) were excluded. ^2 ^ORs from multivariate logistic regression models, adjusted for age, education, gender and religion of parent, and age and gender of the child. ^3 ^The numbers do not add up to the total because of missing answers.

Table 6 shows the associations among students, by gender. The age and religion of the student, and the educational level of their parents were not associated with a propensity to HPV vaccination in either the male or female students. The students who were aware that HPV infection could affect themselves were more in favour of to HPV vaccination, regardless of whether they were male (OR = 5.73, 95% CI 2.85-11.5) or female (OR = 2.39, 95% CI 1.66-3.46). The female students who declared they had a boyfriend (OR = 0.67, 95% CI 0.47-0.97) and had already experienced sexual intercourse (OR = 0.68, 95% CI 0.44-1.03) were less in favour of HPV vaccination, whereas these factors were not associated with the propensity to undergo vaccination among the male students. There was no significant association with a knowledge that HPV vaccine is aimed at preventing cervical cancer.

**Table 6:** Association between selected socio-demographic factors, personal beliefs and characteristics, and attitudes of students to being vaccinated against HPV, by gender.^1^

	Males			Females		
	**In favour of HPV vaccination (n = 78)**	**Against or doubtful about HPV vaccination (n = 123)**	**OR (95% CI) **^**2**^	**In favour of HPV vaccination (n = 431)**	**Against or doubtful about HPV vaccination (n = 196)**	**OR (95% CI) **^**2**^

	**No. (%)**	**No. (%)**		**No. (%)**	**No. (%)**	

**Age (years)**						
<16	29 (37.2)	55 (44.7)	1 (reference)	156 (36.2)	72 (36.7)	1 (reference)
16-17	37 (47.4)	51 (41.5)	1.40 (0.75-2.61)	198 (45.9)	100 (51.0)	0.95 (0.65-1.37)
≥18	12 (15.4)	17 (13.8)	1.38 (0.58-3.31)	77 (17.9)	24 (12.2)	1.51 (0.88-2.59)
**Religion**						
Catholic	66 (84.6)	100 (81.3)	1 (reference)	382 (88.6)	165 (84.2)	1 (reference)
Other/no religion	12 (15.4)	23 (18.7)	0.77 (0.35-1.66)	49 (11.4)	31 (15.8)	0.67 (0.41-1.10)
**Highest education qualification of parents**						
Middle school diploma	7 (9.0)	11 (8.9)	1 (reference)	55 (12.8)	30 (15.3)	1 (reference)
High school diploma	23 (29.5)	38 (30.9)	0.94 (0.32-2.80)	177 (41.1)	82 (41.8)	1.13 (0.67-1.91)
Degree	48 (61.5)	74 (60.2)	1.06 (0.38-2.97)	199 (46.2)	84 (42.9)	1.27 (0.76-2.14)
**Thinks that HPV might concern him/her**^**3**^						
No/Does not know	42 (53.8)	106 (86.2)	1 (reference)	207 (48.7)	136 (69.7)	1 (reference)
Yes	36 (46.1)	17 (13.8)	5.73 (2.85-11.5)	218 (51.3)	59 (30.3)	2.39 (1.66-3.46)
**Has a boyfriend/girlfriend **^**3**^						
No	52 (66.7)	83 (69.7)	1 (reference)	283 (65.8)	113 (57.6)	1 (reference)
Yes	26 (33.3)	36 (30.2)	1.16 (0.62-2.17)	147 (34.2)	83 (42.3)	0.67 (0.47-0.97)
**Has had sexual intercourse **^**3**^						
No	55 (70.5)	90 (76.3)	1 (reference)	335 (78.3)	142 (72.4)	1 (reference)
Yes	23 (29.5)	28 (23.7)	1.30 (0.65-2.62)	93 (21.7)	54 (27.5)	0.68 (0.44-1.03)
**Knows that HPV vaccine is aimed at preventing cervical cancer **^**3**^						
No/Does not know	17 (44.7)	23 (35.9)	1 (reference)	75 (23.1)	36 (28.3)	1 (reference)
Yes	21 (55.3)	41 (64.1)	0.69 (0.30-1.61)	249 (76.8)	91 (71.6)	1.26 (0.79-2.03)

CI = confidence interval; OR = odds ratio. ^1 ^The numbers of subjects are not the same as in Table 4 because those with missing information relating to one or more of the socio-demographic covariates in the models (n = 35; 4% of the total) were excluded. ^2 ^ORs from multivariate logistic regression models, adjusted for age and religion of child, and education of parents. ^3 ^The numbers do not add up to the total because of missing answers.

This study of an upper-middle class population in the greater Milan area, Northern Italy, found that teenagers and parents still have a number of gaps in their knowledge of HPV infection and its prevention by means of vaccination. In particular, they seemed to underestimate the likelihood of HPV infection and were therefore less likely to see HPV vaccination favourably. This is an important indication for future family HPV training programmes designed to increase the acceptance of HPV vaccination.

Most of the parents were aware of HPV and correctly answered a number of questions about it. This might be at least partially explained by their higher education level in Northern Italy in comparison with the general Italian population [27]. There are relatively few other studies of parents, most of which have found a lower level of knowledge of HPV infection. A previous Italian study performed in Southern Italy found that 54% of the mothers of daughters aged 10-12 years had ever heard of HPV [19], and the corresponding figure in a school-based survey conducted in four areas of England was only 26% [28]. However, a US (North Carolina) study reported findings close to ours, with an 83% awareness of HPV among caregivers (mostly parents) and a 69% average of correct answers to questions about it [29].

Nevertheless, a proportion of our parents still need to be better informed about HPV-related health topics. The facts that parents, who are key decision makers in the uptake of this vaccine, are still hesitant to have their daughters receive HPV vaccine, and that strategies to ensure optimal HPV vaccine uptake need to be employed have been recently reported in a population-based evaluation in British Columbia, Canada [30]. In our study, this was especially true for the fathers, whose overall knowledge of HPV (including awareness of HPV infection and its dangerousness, despite in some cases these differences were marginal and not clinically relevant) was significantly less than that of the mothers. Once again, our findings were comparable with those of the North Carolina investigation, which found that only 54% of male caregivers knew about HPV as against 84% of female caregivers [29]. Our findings indicate that interventions aimed at increasing knowledge of HPV should involve the fathers of young children, not least because any health procedure in pediatric subjects must be authorised by both parents in Italy.

Among the students, about 70% of the females and 50% of the males had ever heard of HPV, and about 75% of the former and 66% of the latter know about the aim and mode of HPV vaccination. The data from other studies are mixed [18,31-34]. A cross sectional study of females aged 14-24 years carried out in Southern Italy found that only 30% had ever heard of HPV infection [18], and that the proportion of women who were aware that HPV vaccination is aimed at preventing cervical cancer was also low [18]. In a recent Swedish survey [35], only 5% of the first-year high students of both sexes were aware of HPV, whereas a 1999 study of US university students found that 37% of the respondents had ever heard of it [36].

However, although our findings indicated a better knowledge of HPV among students, it is of concern that only 45% of the female teenagers perceived themselves at risk of HPV infection. This might be explained by a lack of awareness of the high prevalence of HPV infection [9,35], as has been previously found in other studies [37,38]. Furthermore, in addition to having less knowledge of HPV, a large proportion of the male students thought that it did not concern them. This is maybe not surprising as HPV infection and vaccination have almost exclusively been publicised in relation to the prevention of cervical cancer, and it is still being discussed whether males should also be vaccinated [39,40]. Teenagers of both sexes therefore need further information.

We found that the attitude of both parents to vaccinations was very positive, not only because the overwhelming majority were in favour of vaccination in general, but also because about two-thirds said they were in favour of a new vaccination against HPV, even though complete data on the vaccine are not yet available. Fewer than 7% of the parents declared they were certainly against the vaccination, and similarly encouraging results were found among the female students. It is interesting to note that 47% of the parents named pediatricians as their preferred counsellor in the case of decisions concerning vaccinations, and 65% of those with children aged aged <12 years. The recommendation of other physicians (mainly family doctors) was important for 26%, and so these might also play a major role in decision making. In agreement with previous studies [41-45], these data suggest that programmes aimed at increasing vaccination coverage should also involve pediatricians or family physicians depending on the age of the target.

Another finding was that parents consider the indications of the Ministry of Health (48%) rather than Regional institutions (about 3%) as a reference for decision making. This highlights the fact that the general population does not realise that recommendations concerning vaccines (and the availability of free vaccinations) is determined not centrally but at Regional level in Italy.

The main factors associated with the parents' propensity to accept HPV vaccination were: 1) having at least one daughter; 2) being in favour of vaccinations in general; 3) knowing that the vaccine is aimed at preventing cervical cancer; and 4) being aware that their children could be infected by HPV (this association was particularly strong). As also found by other studies [46-49], there was also a positive association between the perceived likelihood of HPV infection and the propensity of both male and female students to undergo vaccination [46-49]. This supports that the first target for the success of HPV vaccination campaign should be to increase adolescents' and parents' knowledge on this topic, particularly underlining the frequency of HPV. Although the association was not statistically significant, our findings also indicated that mothers were more likely to favour having their children vaccinated than fathers. When all of these analyses were limited to the parents of daughters alone, the results were substantially the same.

In addition to their awareness of possible HPV infection, a few other factors were also associated with the propensity of students to undergo vaccination. In particular, having a boyfriend or having already had sexual intercourse were associated with a decreased propensity among female students, possibly because of the high proportion who knew the vaccination should be given before the start of sexual activity. However, this highlights the need for a training programme focused on the epidemiology of HPV infection in different age groups and the risk factors associated with acquiring it, in order to show that vaccination can also be useful even after the beginning of sexual activity provided that the infection has not already been acquired.

The limitations of this study include the fact that our sample was not representative of the general Italian population, recruitment was limited to one Italian Region, a convenience sample of schools was selected and mainly upper-middle class population was included. Though the proportion of respondents to the survey was satisfactorily high, those with higher knowledge and interested by HPV-related health problems were more likely to participate in the study. These considerations are reflected by the high level of education of parents (i.e., about 40% had a university degree, as compared to 24% in the general Milan population [50]) and larger number of female than male students participating to the survey. However, when we corrected our results for educational level of the parents using post-stratification weights [51], the major findings were only marginally different (e.g., the corrected proportion of mothers and fathers who had ever heard of HPV were 88% and 75%, respectively). In addition, we could not obtain information from middle school children (i.e. those aged 10-13 years) because their school boards considered them to be too young. On the contrary, the major strengths of the study are its large size, the availability of concurrent information from both parents and children, and the inclusion of both male and female subjects.

## Conclusions

In conclusion, our data highlight the fact that a better understanding of HPV infection and the possibility of preventing cancer by means of HPV vaccination could increase the acceptance of vaccination by both students and parents. This underlines the need to plan adequate educational programs. Moreover, as physicians (both pediatricians and family physicians) can play a critical role in counseling, their knowledge of HPV infection and its prevention should also be evaluated and improved if necessary.

## List of abbreviations

CI: confidence interval; HPV: human papillomavirus; OR: odds ratio.

## Competing interests

The authors declare that they have no competing interests.

## Authors' contributions

CP, created the database and wrote the manuscript. SE, designed the study and co-wrote the manuscript. CG, performed the statistical analysis. MS, CS, IP, SC, and GM, explained and collected the questionnaires. NP, designed the study and co-wrote the manuscript.

All authors read and approved the final manuscript.

## Pre-publication history

The pre-publication history for this paper can be accessed here:

http://www.biomedcentral.com/1471-2458/10/378/prepub

## Supplementary Material

Additional File 1 **The student questionnaire**. Questionnaire for the adolescents concerning their knowledge on human papillomavirus infection and its prevention. Click here for file

Additional File 2 **The parent questionnaire**. Questionnaire for the parents concerning their knowledge on human papillomavirus infection and its prevention. Click here for file
