# Supplementary material for: Knowledge of human papillomavirus infection and its prevention among adolescents and parents in the greater Milan area, Northern Italy
Source: BMC Public Health. 2010 Jun 28;10:378. doi: 10.1186/1471-2458-10-378 (PMC2901377; doi:10.1186/1471-2458-10-378)
Supplement: Additional File 1 — The student questionnaire. Questionnaire for the adolescents concerning their knowledge on human papillomavirus infection and its prevention. [file 1471-2458-10-378-S1.DOC]

***QUESTIONNAIRE CONCERNING THE KNOWLEDGE ON HUMAN PAPILLOMAVIRUS INFECTION AND ITS PREVENTION***

**ADOLESCENT’S IDENTIFICATION DATA**

Adolescent’s initials: **|__|__|__|**

**A**ge: **|__|__|**

Sex:  M  F

Race  Caucasic

 Middle-oriental

 Hispanica

 Oriental

 Other***____________________________***

Educational qualification of the mother:  Middle school diploma

 High school diploma

 Degree

Educational qualification of the father:  Middle school diploma

 High school diploma

 Degree

Occupation of the mother:  Unemployed

 Housewife

 Blue-collar worker

 White-collar worker

 Manager

 Freelancer

 Other________________________

Occupation of the father:  Unemployed

 Blue-collar worker

 White-collar worker

 Manager

 Freelancer

 Other________________________

Type of occupation of the mother:  Full-time

 Part-time

Type of occupation of the father:  Full-time

 Part-time

Religion:  Catholic

 Jewish

 Moslem

 Other_________________________________

Attendance to religious ceremony:  Always

 Sometimes

 Rarely

 Never

Number of siblings:  F **|__|__|**  M **|__|__|**

Age of siblings:  <10 years, no. **|__|__|**  10-14, no. **|__|__|**

 14-18 years, no. **|__|__|**  >18, no. **|__|__|**

*School:*  ***____________________________________________***

*Data collected by:* ***_____________________________________***

**QUESTIONNAIRE**

**Please, circle only one alternative for each question.**

***Knowledge on HPV infection***

Have you ever heard about HPV?

1. Yes
2. No
3. Do not remember

Do you think that HPV could be dangerous?

# Do not know

# Yes

1. No
2. Only in subjects with chronic diseases

How is transmitted HPV infection?

1. Do not know
2. Sexually
3. With kisses
4. With foods
5. Other, specify ________________

Have you ever heard of HPV vaccination?

1. Do not remember
2. No
3. Yes

If yes, which is the main aim of HPV vaccination?

1. Do not know
2. Prevention of cervical cancer
3. Prevention of pregnancy
4. Prevention of a sexually transmitted disease
5. Other, specify_________________________________________

If yes, from which source?

1. Paediatrician
2. Other physician
3. Parents
4. Teachers
5. Friends
6. TV/radio
7. Internet
8. Other, specify _________________________________

***Knowledge and personal attitudes towards HPV vaccination***

Do you think HPV infection might concern you?

# Do not know

# Yes

1. No

Do you want to perform HPV vaccination?

1. Do not know
2. No
3. Yes

If you do not want to perform HPV vaccination, why do you give this answer?

1. I do not know what it is aimed to
2. I am a male and only females should be vaccinated
3. Fear to execute an injection
4. No fear of the illness related to HPV
5. Fear of vaccine– related adverse events
6. Religious reasons
7. Other, explain______________________________________________

If you want to perform HPV vaccination, why do you give this answer?

- 1. Prevention of a sexually transmitted disease
  2. Prevention of a potentially carcinogenic infection
  3. Other, specify________________________________________

Where would you like to be vaccinated against HPV?

- - 1. Paediatrician
    2. Gynecologist
    3. Other physician
    4. Local Health Unit
    5. School
    6. Hospital

When is HPV vaccination recommended?

1. Do not know
2. Within the first year of life
3. Before the beginning of sexual activity
4. After the beginning of sexual activity
5. When a pregnancy is planned
6. Other, specify ____________________________________________

At what age do you think that should be given information about the possibility of HPV prevention?

1. Do not know
2. 10-13 years
3. 14-18 years
4. 18-30 years
5. >30 years

Do you require more information on HPV and its prevention?

- 1. Do not know
  2. No
  3. Yes

If you do not think to require more information on HPV and its prevention, why do you give this answer?

1. HPV and its prevention do not interest me
2. Never had sexual intercourses
3. I always use condoms
4. This topic is not relevant
5. Other, specify_____________________________________________

If you require more information on HPV and its prevention, who is the person that should give you information?

1. Paediatrician or general medicine doctor
2. Teacher at school
3. Other, specify_______________________________________

***Sexual activity and attitudes towards discussing problems related to sexuality***

Do you have a boy/girl friend?

1. Yes
2. No

Did you have at least once sexual intercourse?

1. Yes
2. No

If yes, at which age did you have the first one?

Specificy__________yrs

If yes, with how many persons have you had sexual intercourses?

1. One
2. Two
3. Three
4. More than three

Do you use contraceptive methods?

1. No
2. Yes

If yes, which ones?

1. Condom
2. Pill
3. Other, specify_________________

Who do you talk about questions related to sexuality?

1. Friends
2. Parents
3. Teachers
4. Religious
5. Paediatrician
6. Family physician
7. Gynecologist
8. Other, specify ________________________________________

Do you talk about questions related to sexuality with your parents?

1. Never
2. Only if they begin to speech
3. Only if I have specific problems
4. Always

Do you talk about sexually transmitted diseases with your parents?

- 1. Never
  2. Only if they begin to speech
  3. Only if I have specific problems
  4. Always
