# Supplementary material for: Knowledge of human papillomavirus infection and its prevention among adolescents and parents in the greater Milan area, Northern Italy
Source: BMC Public Health. 2010 Jun 28;10:378. doi: 10.1186/1471-2458-10-378 (PMC2901377; doi:10.1186/1471-2458-10-378)
Supplement: Additional File 2 — The parent questionnaire. Questionnaire for the parents concerning their knowledge on human papillomavirus infection and its prevention. [file 1471-2458-10-378-S2.DOC]

***QUESTIONNAIRE CONCERNING THE KNOWLEDGE ON HUMAN PAPILLOMAVIRUS INFECTION AND ITS PREVENTION***

**PARENT’S IDENTIFICATION DATA**

Parent’s initials: **|__|__|__|**

**A**ge: **|__|__|**

Sex:  M  F

Race  Caucasic

 Middle-oriental

 Hispanica

 Oriental

 Other***____________________________***

Educational qualifications:  Medium school

 High-level school

 Degree

Occupation?  Unemployed

 Housewife

 Blue-collar worker

 White-collar worker

 Manager

 Freelancer

 Other________________________

Type of occupation:  Full-time

 Part-time

Religion:  Catholic

 Jewish

 Moslem

 Other_________________________________

Attendance to religious ceremony:  Always

 Sometimes

 Rarely

 Never

Number of sons/daughters: **|__|__|**

Age of sons/daughters attending the school investigated: **|__|__|**

**|__|__|**

**|__|__|**

Gender of offspring attending the school investigated:

 M, no.___________  F, no.__________________

Is there someone in your family with a history of cancer?

 No

 Yes

Is there someone in your family with a history of uterine cancer?

 No

 Yes

***Attitudes towards vaccination in general***

Are you in favour of vaccinations in general?

 No

 Yes

If no, why?  They are not always useful

 Because of potential adverse effects

 They might cause problems

 It is better to have the disease

 Other, specify ____________________

Why do you vaccinate your sons/daughters?

 It is compulsory

 Suggestion of the paediatrician

 Suggestion of the family physician

 I gather information on newspapers/TV

 I gather information on the Internet

 Other, specify _____________________

Which recommendation is significant to you to decide on a vaccination for your sons/daughters?

 Ministry of Health

 Regional Body

 Paediatrician

 Other physician

 Relative/friend

 Teacher

 Religious authority

 Altro, specificare_____________________

*School:*  ***____________________________________________***

*Data collected by:* ***_____________________________________***

**QUESTIONNAIRE**

**Please, circle only one alternative for each question.**

***Knowledge on HPV and HPV-related diseases***

Have you ever heard about HPV?

1. Yes
2. No

Do you think that HPV could be dangerous?

# Do not know

# Yes

1. No
2. Only in subjects with chronic diseases

How is transmitted HPV infection?

1. Do not know
2. Sexually
3. With strict contact
4. With foods
5. Other, specify ________________

Which is the main aim of HPV vaccination?

1. Do not know
2. Prevention of cervical cancer
3. Prevention of pregnancy
4. Prevention of a sexually transmitted disease
5. Other, specify_________________________________________

Do you think that HPV could involve your sons/daughters?

1. Do not know
2. Yes
3. No

If no, why?

1. They have no sexual intercourses yet
2. They do not have sexual intercourses at risk
3. They have no family history of cancer
4. Other, specify_____________________________________________

***Knowledge and personal attitudes towards HPV vaccination***

Do you want that your sons/daughthers perform HPV vaccination?

1. No
2. Yes
3. Maybe / not sure

If you want that your sons and/or daughthers perform HPV vaccination, why do you give this answer?

- 1. Prevention of a sexually transmitted disease
  2. Prevention of a potentially carcinogenic infection
  3. Other, specify________________________________________

If you do not want that your sons and daughters perform HPV vaccination, why do you give this answer?

1. Fear to execute an injection to my son/daughter
2. No fear of the illness
3. Fear of vaccine– related adverse events
4. Refusal of a vaccination which prevents a sexually transmitted illness
5. Religious reasons
6. Fear that the vaccination may encourage sexual activity
7. Other, explain______________________________________________

If you want that your sons and/or daughters perform HPV vaccination, which is the ideal age for giving them the vaccine?

- - 1. Do not know
    2. 10-11 years
    3. 12-13 years
    4. 14-18 years
    5. >18 years

When is HPV vaccination recommended?

1. Do not know
2. Within the first year of life
3. Before the beginning of sexual activity
4. After the beginning of sexual activity
5. When a pregnancy is planned
6. Other, explain ____________________________________________

Do you require more information on HPV and its prevention?

- 1. No
  2. Yes

If you do not think to require more information on HPV and its prevention, why do you give this answer?

1. HPV and its prevention do not interest me and my children
2. This topic is not relevant
3. Other, specify_____________________________________________

If you require more information on HPV and its prevention, which is the best way system to increase your knowledge?

1. Simple and clear books in my language on this argument
2. Specific discussions with physicians of different specialities
3. Other, explain_____________________________________________

At what age do you think that should be given information about the possibility of HPV prevention?

1. I do not know
2. 10-11 years
3. 12-13 years
4. 14-18 years
5. >18 years

***Attitudes of discussing problems related to sexuality with sons/daughters***

Do you talk about questions related to sexuality with your sons/daughters?

1. Never
2. Only if they begin to speech
3. Only if they have specific problems
4. Always

Do you talk about sexually transmitted diseases with your sons/daughters?

- 1. Never
  2. Only if they begin to speech
  3. Only if they have specific problems
  4. Always

Do you think that your sons/daughters have to be informed on HPV and its prevention?

1. Yes
2. No

If you think that your sons/daughters have not to be informed on HPV and its prevention, why do you give this answer?

1. This topic is not interesting for them
2. Parents have to decide for their children
3. Religious reasons
4. Other, specify_________________________________________

If you think that your sons/daughters have to be informed on HPV and its prevention, who should give them information?

1. Paediatrician / Family physician
2. Teacher at school
3. Other, specify_______________________________________
